# Author Correction: Personalized aerosolised bacteriophage treatment of a chronic lung infection due to multidrug-resistant *Pseudomonas aeruginosa*

**DOI:** 10.1038/s41467-023-40202-3

**Published:** 2023-07-24

**Authors:** Thilo Köhler, Alexandre Luscher, Léna Falconnet, Grégory Resch, Robert McBride, Quynh-Anh Mai, Juliette L. Simonin, Marc Chanson, Bohumil Maco, Raphaël Galiotto, Arnaud Riat, Natacha Civic, Mylène Docquier, Shawna McCallin, Benjamin Chan, Christian van Delden

**Affiliations:** 1 Service of Infectious Diseases, Geneva University Hospitals, Geneva, Switzerland; 2 Department of Microbiology and Molecular Medicine, University of Geneva, Geneva, Switzerland; 3 Center for Research and Innovation in Clinical Pharmaceutical Sciences (CRISP), Lausanne University Hospital (CHUV), Lausanne, Switzerland; 4 Felix Biotechnology, San Francisco, CA USA; 5 Department of Cell Physiology and Metabolism, University of Geneva, Geneva, Switzerland; 6 Diagnostic Bacteriology Laboratory, Geneva University Hospitals, Geneva, Switzerland; 7 iGE3 Genomics Platform, University of Geneva, Geneva, Switzerland; 8 Department of Neuro-Urology Balgrist Hospital, Zurich, Switzerland; 9 Yale University, New Haven, CT USA

**Keywords:** Translational research, Bacteriophages

Correction to: *Nature Communications* 10.1038/s41467-023-39370-z, published online 27 June 2023

Post-publication, authors requested that two additional authors should be added to the author list. This has been approved by all corresponding authors. The previous author list and the modified author list are noted below.

Previous author list:

Thilo Köhler^1,2^*, Alexandre Luscher^1,2^, Léna Falconnet^1,2^, Grégory Resch^3^, Robert McBride^4^, Quynh-Anh Mai^4^, Juliette L. Simonin^5^, Marc Chanson^5^, Bohumil Maco^2^, Raphaël Galiotto^2^, Arnaud Riat^6^, Shawna McCallin^7^, Benjamin Chan^8^ and Christian van Delden^1,2^

Modified author list:

Thilo Köhler^1,2^*, Alexandre Luscher^1,2^, Léna Falconnet^1,2^, Grégory Resch^3^, Robert McBride^4^, Quynh-Anh Mai^4^, Juliette L. Simonin^5^, Marc Chanson^5^, Bohumil Maco^2^, Raphaël Galiotto^2^, Arnaud Riat^6^, Natacha Civic^7^, Mylène Docquier^7^, Shawna McCallin^8^, Benjamin Chan^9^ and Christian van Delden^1,2^

^7^iGE3 Genomics Platform, University of Geneva, 1211, Geneva, Switzerland.

