# Supplementary material for: Personalized aerosolised bacteriophage treatment of a chronic lung infection due to multidrug-resistant Pseudomonas aeruginosa
Source: Nat Commun. 2023 Jun 27;14:3629. doi: 10.1038/s41467-023-39370-z (PMC10300124; doi:10.1038/s41467-023-39370-z)
Supplement: Supplementary file 1 — Supplementary information [file 41467_2023_39370_MOESM1_ESM.pdf]

Table S1. Susceptibility of *P. aeruginosa* isolates to phage vFB297 and to antibiotics

Strain/Isolate	Susceptibility Φ vFB297	MIC (mg/L)			
		AZM	CAZ	MER	CIP
PAO1	S	4	2	0.5	0.125
**D-100**	S	>32	>32	>16	8
**D0.1**	R	0.25	2	2	8
D0.2	S	2	8	8	8
D0.3	S	4	8	8	8
D0.4	S	8	8	8	8
D0.5	R	0.5	4	>16	8
D0.6	I	>32	>32	>16	8
D1.1	S	1	8	16	8
D1.2	S	>32	>32	>16	4
D1.3	S	4	8	>16	8
D1.4	S	0.5	16	>16	ND
D2.1	S	8	32	>16	4
D2.2	S	>32	>32	>16	8
D2.3	S	16	32	>16	8
D3.1	S	16	16	>16	4
D3.2	S	>32	8	4	8
D3.3	S	0.5	32	>16	8
D4.1	S	>32	>32	>16	16
D4.2	S	4	4	4	4
D4.3	S	>32	>32	>16	16
**D6.1**	S	4	8	4	8
D6.2	S	32	32	16	8
D6.3	S	4	8	8	8
**D75.1**	S	32	>32	16	16
**D315.1**	S	32	>32	8	ND
**D455**	S	32	>32	8	ND
**D467**	S	>32	>32	8	ND

S, susceptible (plaques visible on phage dilutions); I, intermediate (plaques visible only with undiluted phage); R; resistant (no plaques) AZM, aztreonam; CAZ, ceftazidime; MER, meropenem; CIP, ciprofloxacin; sequenced isolates are shown in bold. Phage treatment periods are shaded in grey. ND, not determined.

Table S2. Primers used in this study.

Name	Purpose	Sequence (5'-3')
**ampC1**	*P. aeruginosa* qPCR	CGGCTCGGTGAGCAAGACCTTC
**ampC2**	*P. aeruginosa* qPCR	AGTCGCGGATCTGTGCCTGGTC
**rpsL-F**	*P. aeruginosa* gDNA detection/normalization	GCAAGCGCATGGTCGACAAGA
**rpsL-R**	*P. aeruginosa* gDNA detection/normalization	CGCTGTGCTCTTGCAGGTTGTG
**AX-F1**	*A. xylosoxidans* gDNA detection	GTCTACGCCTATGACCTGTTCC
**AX-R1**	*A. xylosoxidans* gDNA detection	GTCTCGATCATGTCGAACTGC
**SP-Phi30.1**	Phage vFB297 gDNA detection	ATAGTCCGATCATCGCCTTCT
**SP-Phi30.2**	Phage vFB297 gDNA detection	GTAGAGCCTGTGCTTCCTCCT
**ms172-forward**	DLST genotyping	GGATTCTCTCGCACGAGGT
**ms172-reverse**	DLST genotyping	TACGTGACCTGACGTTGGTG
**ms217-forward**	DLST genotyping	TTCTGGCTGTCGCGACTGAT
**ms217-reverse**	DLST genotyping	GAACAGCGTCTTTTCCTCGC

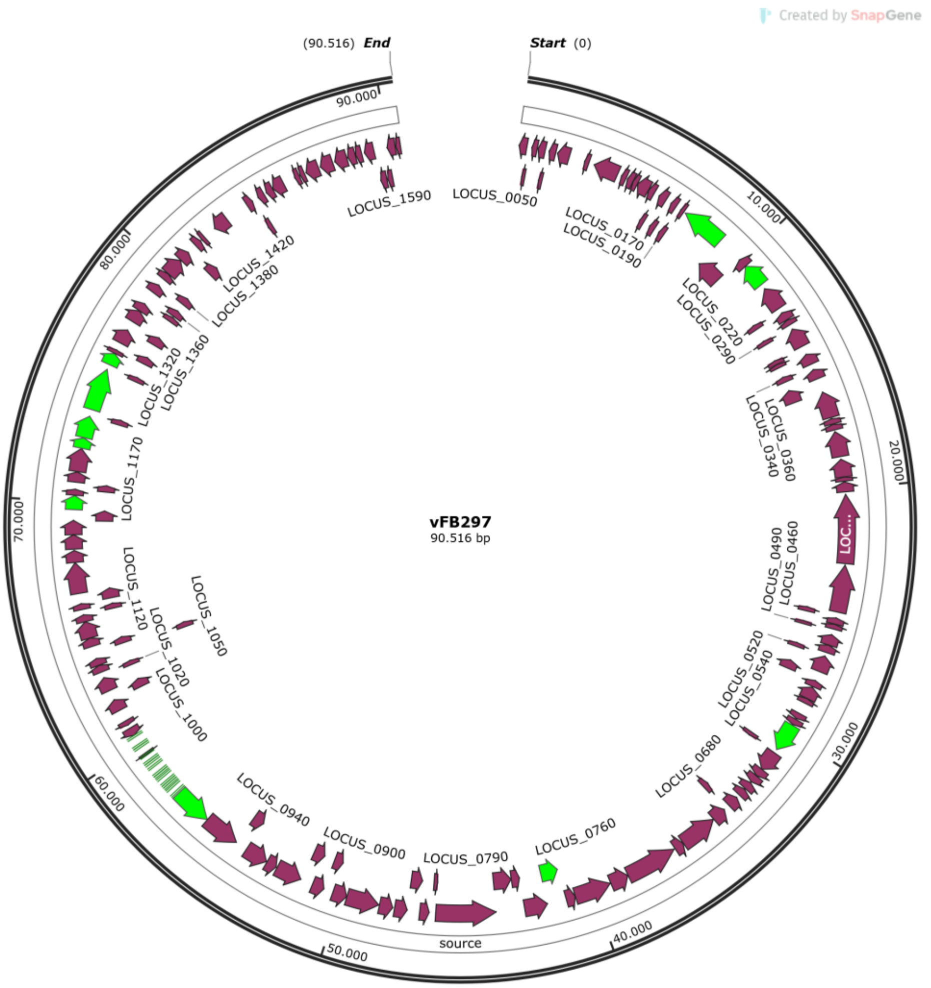

Fig. S1. **Genomic map of phage vFB297**. BLAST search in the NCBI database retrieved Pseudomonas phages 908-1, SRT6, PaYy-2 and vB_PaeM_SCUT-S2 as showing more than 94% nucleotide identity with vFB297. These phages are annotated as belonging to the genus *Pakpunavirus*. Their complete genome sizes (90 – 95 kbp) are comparable to those of phage vFB297 (90,516 bp). The genome sequence of vFB297 was annotated using DFAST (https://dfast.ddbj.nig.ac.jp/help_annotation) and the genome map generated with SnapGene. Arrows in green identify genes with functional predictions, while all other arrows indicate genes encoding hypothetical proteins. The phage genome was submitted to NCBI GenBank accession # OQ921398 (https://www.ncbi.nlm.nih.gov/nuccore/OQ921398).

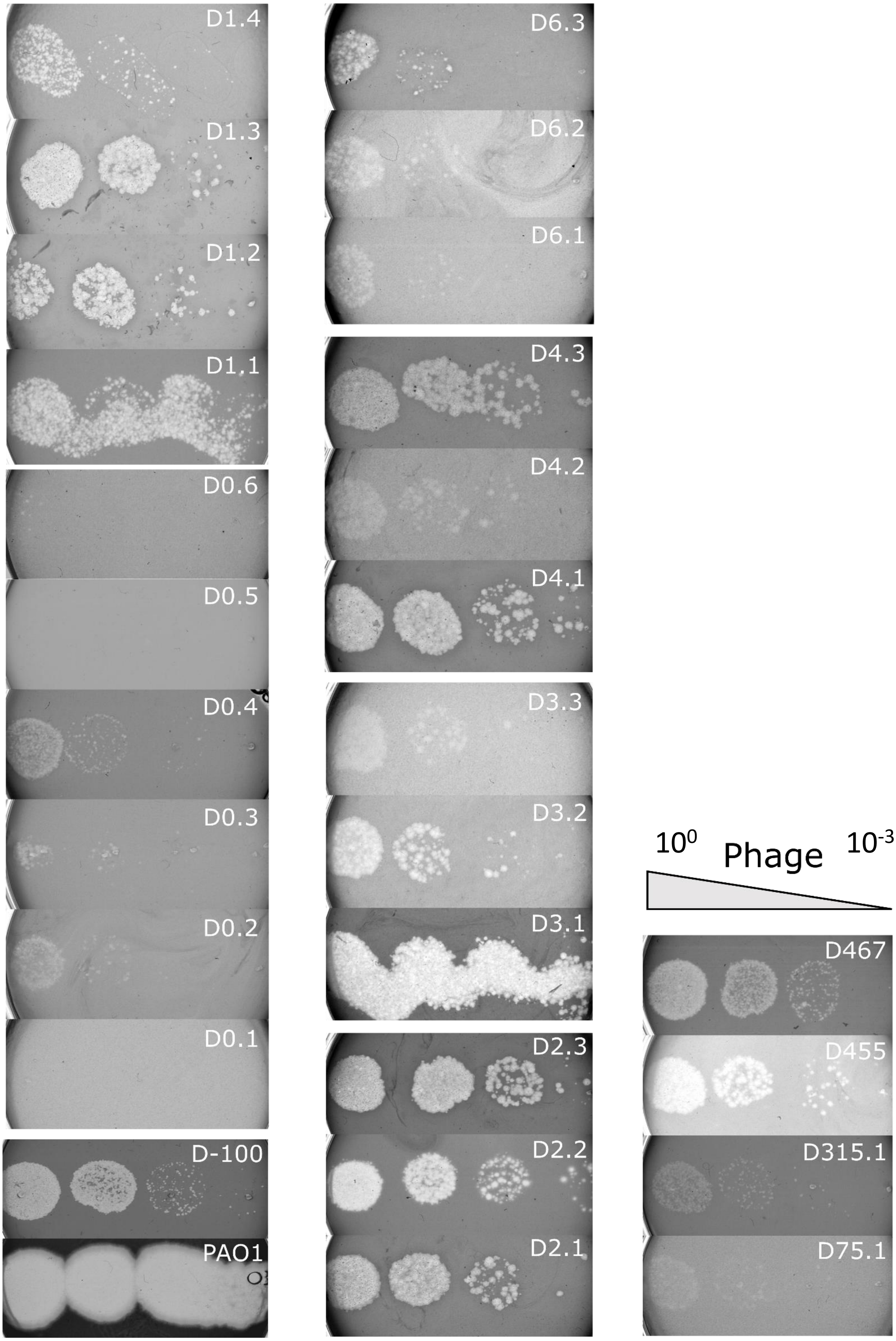

Fig. S2. **Susceptibility of patient isolates to phage vFB297**. Each isolate was tested by double agar overlay assays for susceptibility to serial 10-fold dilutions of phage vFB297 prepared in NaCl 0.9%. Plates were scored after 20 h growth at 37 °C. Only isolates D0.1 and D0.5 produced no visible plaques in this assay.

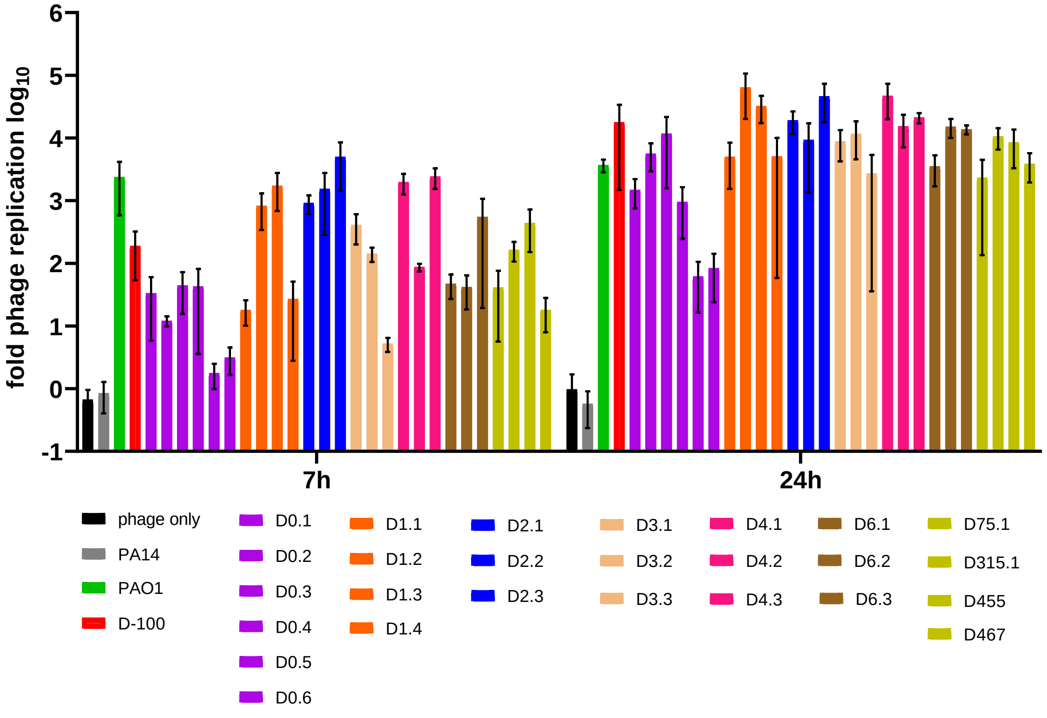

Fig. S3. Replication of phage vFB297 on patient isolates. PAO1 and PA14 as well as patient isolates were tested for phage replication at 7 h and 24 h after phage addition. PFU were determined on PAO1 bacteria after growth at 37 °C for 20 h. Isolates D0.5 and D0.6, considered as resistant and intermediate respectively, according to plaque assays (Fig. S1), showed the lowest phage replication. PA14, resistant to phage vFB297, was included as a control strain (grey bars). Values are mean ± SEM of three independent biological replicates. Source data are provided with this paper.

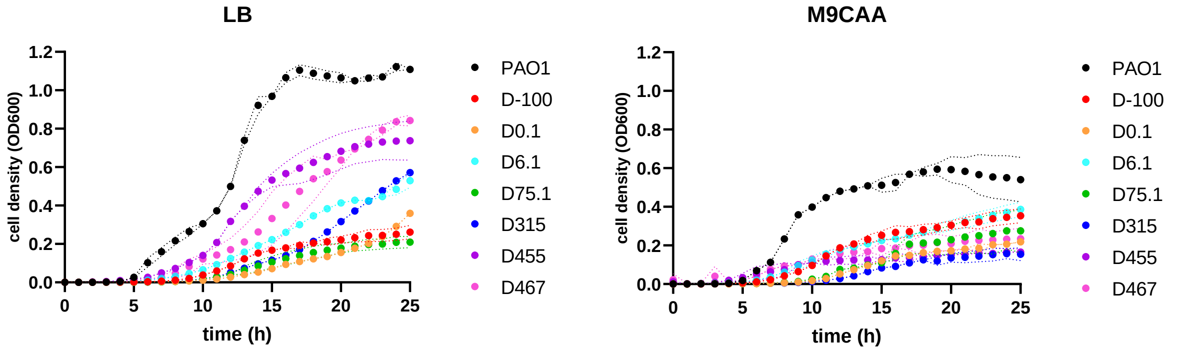

Fig. S4. **Growth curves of *P. aeruginosa* patient isolates.** Growth was determined in (A) LB and in (B) iron-limited M9 casamino acids medium (M9CAA) at 37 °C in microtiter plates. Data are the average and standard deviations (stippled lines) of duplicate determinations performed on two separate occasions. Source data are provided with this paper.
