# Supplementary material for: Personalized aerosolised bacteriophage treatment of a chronic lung infection due to multidrug-resistant Pseudomonas aeruginosa
Source: Nat Commun. 2023 Jun 27;14:3629. doi: 10.1038/s41467-023-39370-z (PMC10300124; doi:10.1038/s41467-023-39370-z)
Supplement: Supplementary file 3 — Description of Additional Supplementary Files [file 41467_2023_39370_MOESM3_ESM.pdf]

<u>Description of Additional Supplementary Files</u>

Supplementary dataset 1: SNP and InDels of seven sequenced clinical isolates based on alignment with genomic sequence of strain PA14 (NC_008463.1)

Supplementary dataset 2: Large deletions in seven sequenced clinical isolates based on alignment with genomic sequence of strain PA14 (NC_008463.1)
