# Supplementary material for: Personalized aerosolised bacteriophage treatment of a chronic lung infection due to multidrug-resistant Pseudomonas aeruginosa
Source: Nat Commun. 2023 Jun 27;14:3629. doi: 10.1038/s41467-023-39370-z (PMC10300124; doi:10.1038/s41467-023-39370-z)
Supplement: Supplementary file 6 — Reporting Summary [file 41467_2023_39370_MOESM6_ESM.pdf]

nature portfolio

Corresponding author(s): Thilo Köhler

Last updated by author(s): 05.06.2023

Reporting Summary

Nature Portfolio wishes to improve the reproducibility of the work that we publish. This form provides structure for consistency and transparency in reporting. For further information on Nature Portfolio policies, see our Editorial Policies and the Editorial Policy Checklist.

Statistics

For all statistical analyses, confirm that the following items are present in the figure legend, table legend, main text, or Methods section.

n/a	Confirmed	
☐	☒	The exact sample size (*n*) for each experimental group/condition, given as a discrete number and unit of measurement
☐	☒	A statement on whether measurements were taken from distinct samples or whether the same sample was measured repeatedly
☐	☒	The statistical test(s) used AND whether they are one- or two-sided<br>*Only common tests should be described solely by name; describe more complex techniques in the Methods section.*
☒	☐	A description of all covariates tested
☐	☒	A description of any assumptions or corrections, such as tests of normality and adjustment for multiple comparisons
☐	☒	A full description of the statistical parameters including central tendency (e.g. means) or other basic estimates (e.g. regression coefficient) AND variation (e.g. standard deviation) or associated estimates of uncertainty (e.g. confidence intervals)
☐	☒	For null hypothesis testing, the test statistic (e.g. *F*, *t*, *r*) with confidence intervals, effect sizes, degrees of freedom and *P* value noted<br>*Give P values as exact values whenever suitable.*
☒	☐	For Bayesian analysis, information on the choice of priors and Markov chain Monte Carlo settings
☒	☐	For hierarchical and complex designs, identification of the appropriate level for tests and full reporting of outcomes
☒	☐	Estimates of effect sizes (e.g. Cohen's *d*, Pearson's *r*), indicating how they were calculated

Our web collection on statistics for biologists contains articles on many of the points above.

Software and code

Policy information about availability of computer code

Data collection	Graphpad prism 9.4.0 & 9.5.1, Microsoft excel, iTEM
Data analysis	Graphpad prism 9.4.0 & 9.5.1, vcf2phylip v2.6, imageJ v1.53

For manuscripts utilizing custom algorithms or software that are central to the research but not yet described in published literature, software must be made available to editors and reviewers. We strongly encourage code deposition in a community repository (e.g. GitHub). See the Nature Portfolio guidelines for submitting code & software for further information.

Data

Policy information about availability of data

All manuscripts must include a data availability statement. This statement should provide the following information, where applicable:

- Accession codes, unique identifiers, or web links for publicly available datasets
- A description of any restrictions on data availability
- For clinical datasets or third party data, please ensure that the statement adheres to our policy

The raw data generated in this study have been deposited to Zenodo: doi: 10.5281/zenodo.8005315 (https://zenodo.org/search?page=1&size=20&q=8005315). The phage sequence has been submitted to GenBank under the accession number #OQ921398 (https://www.ncbi.nlm.nih.gov/nuccore/OQ921398). Genomic sequencing reads of the patient isolates are available at the Sequence Read Archive (SRA) BioProject PRJNA980116 (http://www.ncbi.nlm.nih.gov/bioproject/8980116). Source data are provided with this paper.

Research involving human participants, their data, or biological material

Policy information about studies with human participants or human data. See also policy information about sex, gender (identity/presentation), and sexual orientation and race, ethnicity and racism.

Reporting on sex and gender	This is a case report describing one male patient and its samples.
Reporting on race, ethnicity, or other socially relevant groupings	As n=1, no relevant groupings are possible
Population characteristics	This study describes the case of a 41-year old male, caucasian
Recruitment	Participant was selected as being eligible for compassionate use of experimental phage treatment. The self-selection bias is not applicable since no other therapeutic options were available, leading the authors to offer an experimental treatment to the patient.
Ethics oversight	Geneva University Hospitals, Geneva , Switzerland

Note that full information on the approval of the study protocol must also be provided in the manuscript.

Field-specific reporting

Please select the one below that is the best fit for your research. If you are not sure, read the appropriate sections before making your selection.

☒ Life sciences ☐ Behavioural & social sciences ☐ Ecological, evolutionary & environmental sciences

For a reference copy of the document with all sections, see nature.com/documents/nr-reporting-summary-flat.pdf

Life sciences study design

All studies must disclose on these points even when the disclosure is negative.

Sample size	Samples were collected when possible, i.e. when the patient yielded produtive expectorates during a hospital stay
Data exclusions	No data were excluded
Replication	Experiments were performed in replicates and at multiple occasions. Depending on the experiment type, 2 to 3 independent experiments were performed on different days. Replications were successful.
Randomization	Not relevant for our study as all samples were taken from one single patient at different time-points, thus group allocations and randomizations were not applicable
Blinding	Blinding was not relevant to our study as all samples come from one single patient.

Reporting for specific materials, systems and methods

We require information from authors about some types of materials, experimental systems and methods used in many studies. Here, indicate whether each material, system or method listed is relevant to your study. If you are not sure if a list item applies to your research, read the appropriate section before selecting a response.

Materials & experimental systems

n/a	Involved in the study	
☒	☐	Antibodies
☐	☒	Eukaryotic cell lines
☒	☐	Palaeontology and archaeology
☒	☐	Animals and other organisms
☒	☐	Clinical data
☒	☐	Dual use research of concern
☒	☐	Plants

Methods

n/a	Involved in the study	
☒	☐	ChIP-seq
☒	☐	Flow cytometry
☒	☐	MRI-based neuroimaging

Eukaryotic cell lines

Policy information about cell lines and Sex and Gender in Research

Cell line source(s)	Calu-3 cells, also called HTB-55<br>Fogh J. Human tumor cells in vitro. New York: Plenum Press; 1975.<br>ATCC,Manassas, VA, United States<br>CVCL_0609
Authentication	As cell line was obtained from ATCC, the cell line was not further authenticated
Mycoplasma contamination	Cell line was not tested for mycoplasma contamination
Commonly misidentified lines<br>(See ICLAC register)	The used cell line is not part of the list of misidentified cell lines
